# Protocol for identifying cellular reprogramming minimal networks using combinatorial transcription factor screening

**DOI:** 10.1016/j.xpro.2026.104527

**Published:** 2026-04-28

**Authors:** Abigail R. Altman, Diogo Pértiga-Cabral, Carlos-Filipe Pereira, Ilia Kurochkin

**Affiliations:** 1 Molecular Medicine and Gene Therapy, Science for Life Laboratory, Lund Stem Cell Center, Lund University, BMC A12, 221 84 Lund, Sweden; 2 Wallenberg Centre for Molecular Medicine, Lund University, Lund, Sweden; 3 CNC - Centre for Neuroscience and Cell Biology, University of Coimbra, Largo Marquês Do Pombal, 3004-517 Coimbra, Portugal; 4 CiBB - Centre for Innovative Biomedicine and Biotechnology, University of Coimbra, Coimbra, Portugal; 5 Doctoral Programme in Experimental Biology and Biomedicine, University of Coimbra, Coimbra, Portugal; 6 Asgard Therapeutics AB, Medicon Village, 223 81 Lund, Sweden

**Keywords:** Bioinformatics, Single Cell, Flow Cytometry, Sequencing, RNAseq, Immunology, Stem Cells

## Abstract

Direct reprogramming offers a powerful approach to generate therapeutic cell types, but progress is limited by an incomplete understanding of transcription factor (TF) cooperativity. Here, we present a protocol for performing combinatorial TF screening to resolve reprogramming factor networks that drive cell identity. We describe steps for arrayed lentiviral production, transduction, and reprogramming of human fibroblasts into distinct immune cells. We detail procedures for cell purification, library preparation, sequencing, and analysis to resolve TF combinations and dynamics.

For complete details on the use and execution of this protocol, please refer to Kurochkin et al.^1^

## Highlights

• Comprehensive protocol for producing arrayed lentiviral TF libraries
• Guidance for optimizing reprogramming and achieving uniform TF distribution
• Strategy for purifying cells and generating single-cell transcriptomes and barcodes
• Bioinformatics pipeline for detecting barcodes and assembling TF combinations

## Abstract (editor highlights)

Publisher’s note: Undertaking any experimental protocol requires adherence to local institutional guidelines for laboratory safety and ethics.

## Graphical abstract

**Figure:**
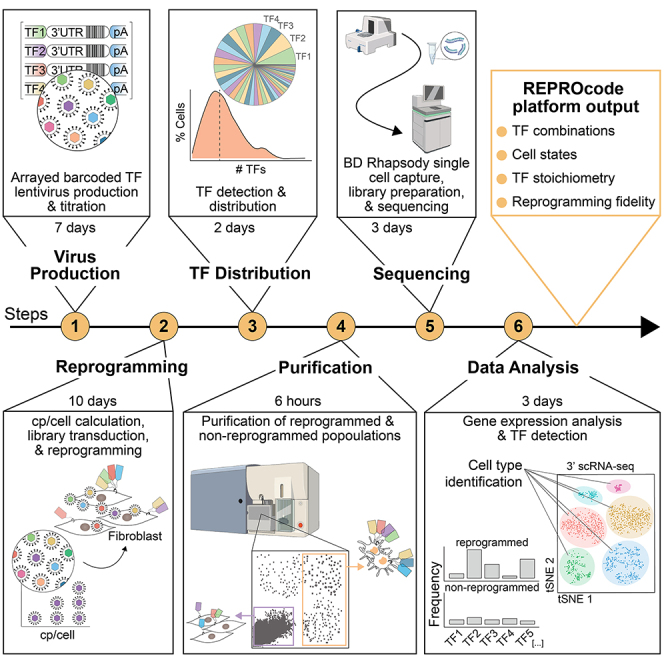

## Before you begin

The protocol below describes the specific steps for combinatorial transcription factor (TF) screening in human embryonic fibroblasts (HEFs). This approach is also applicable to other adherent cell types, including mouse and human cell lines; however, optimization for each cell type and experimental context is strongly recommended. Users define the target cell fates and select candidate TFs accordingly. TFs can be chosen from the REPROcode library, which provides 408 ready-to-use immune-restricted TFs.

Lentivirus production must be performed in a Biosafety Level 2 (BSL-2) cell culture laboratory using a designated, lentiviral-dedicated laminar flow hood. Serological pipettes, pipette tips, tissue culture-treated dishes, and other lentiviral-contaminated consumables must be disposed of according to institutional guidelines.

Lentiviral titration and single-cell library preparation require work areas and equipment free from contaminating nucleic acids.

This protocol describes single-cell capture, cDNA synthesis, and library preparation using the BD Rhapsody System and has also been validated using the 10X Genomics Chromium platform.

### Innovation

The protocol presented here introduces a combinatorial, barcoded TF screening platform for cellular reprogramming that enables systematic dissection of cell fate–instructive TF networks at single-cell resolution. Classical approaches for identifying instructive TFs include iterative *n*-1 dropout screens,^2^^,^^3^ anchored screening strategies,^4^ and *in silico* predictions of gene regulatory networks.^5^^,^^6^^,^^7^^,^^8^ Although recent advances in single-cell barcoding technologies have increased scalability for larger TF libraries,^9^^,^^10^^,^^11^^,^^12^ these methods typically assess the effects of individual TFs and fail to capture the minimal combinatorial networks required for effective transdifferentiation.^13^ Unlike existing reprogramming approaches that test limited TF combinations, rely on trial-and-error optimization, or evaluate single TF activity in single cells, REPROcode enables simultaneous interrogation of TF combinations, relative stoichiometry, and dynamic regulators of reprogramming within a single experiment.

The innovation of this protocol lies in the integration of an arrayed lentiviral library design with single-cell transcriptomics to resolve combinatorial TF effects in a scalable and quantitative manner. The workflow is optimized for immune cell reprogramming but remains adaptable to other lineages. Importantly, this protocol enables barcode detection directly from whole-transcriptome sequencing data, eliminating the need for barcode-specific amplification and reducing experimental complexity and bias. In addition, the protocol provides a customizable computational pipeline that links barcode recovery with transcriptional state assignment, enabling reconstruction of TF combinations, higher-order meta-combinations, and reprogramming trajectories. Beyond identification of minimal instructive TF networks, REPROcode allows for the discovery of additional regulators that enhance reprogramming fidelity by examining lineage-affiliated cells that co-express a core TF combination together with supplementary TFs. Together, these innovations merge experimental and computational strategies into a unified workflow that advances reprogramming methodologies and provides a framework for decoding and engineering cell-fate decisions across diverse biological contexts.

## Key resources table

**Table:** 

REAGENT or RESOURCE	SOURCE	IDENTIFIER
**Antibodies**		

PE anti-human CD45 Antibody	Biolegend	Cat #304008; RRID: AB_314396
APC anti-human HLA-DR Antibody	Biolegend	Cat #307610; RRID: AB_314688

**Chemicals, peptides, and recombinant proteins**		

HyClone Dulbecco’s Modified Eagle Medium (DMEM) high glucose with L-glutamine, sodium pyruvate	Cytiva	Cat #SH30243.01
GlutaMAX Supplement	Gibco	Cat #35050061
Opti-MEM Reduced Serum Medium	Gibco	Cat #31985070
Fetal Bovine Serum (FBS)	Gibco	Cat #A5256701
HyClone™ Penicillin-Streptomycin (Pen-Strep) 100X solution	Cytivia	Cat #SV30010
Dulbecco’s Phosphate Buffered Saline (PBS), 1X, without Calcium, Magnesium	Gibco	Cat #SH30028
Sodium butyrate	Sigma-Aldrich	Cat #B5887
Polybrene	Sigma-Aldrich	Cat #TR-1003-G
Polyethylenimine (PEI)	Sigma-Aldrich	Cat #408727
10% Bovine Serum Albumin (BSA)	Stem Cell Technologies	Cat #09300
EDTA, 0.5 M, pH 8.0	Invitrogen	Cat #AM9260G
Gelatin from porcine skin	Sigma-Aldrich	Cat #G2500
TrypLE™ Express Enzyme (1X)	Gibco	Cat #12604021
Mouse serum	Sigma-Aldrich	Cat #M5905
DAPI (4′,6-Diamidino-2-phenylindole dihydrochloride)	Sigma-Aldrich	Cat #D9542

**Critical commercial assays**		

Lenti-X Concentrator	Takara	Cat #631232
NucleoSpin RNA Virus, Minikit for viral RNA/DNA	Macherey-Nagel	Cat #740956
Lenti-X qRT-PCR titration kit	Takara	Cat #631235
Rhapsody 8 Lane Cartridge	BD Biosciences	Cat #666262
BD Rhapsody Enhanced Cartridge Reagent Kit V3	BD Biosciences	Cat #667052
Rhapsody cDNA Kit	BD Biosciences	Cat #633773
BD Rhapsody WTA Amplification Kit	BD Biosciences	Cat #633801
Rhapsody Targeted mRNA & AbSeq Amp Kit	BD Biosciences	Cat #633774
Rhapsody Custom Panel 2–99 Genes/TARGET	BD Biosciences	Cat # 633777

**Experimental models: Cell lines**		

HEK293T	ATCC	Cat #CRL-3216
Human embryonic fibroblasts (HEF)	Kurochkin *et al.* 2026^1^	N/A
YUMM1.7	Ascic *et al.* 2024^14^	N/A

**Oligonucleotides**		

Barcode targeted primers (N1 and N2)	Kurochkin *et al.* 2026^1^	N/A

**Recombinant DNA**		

psPAX2	Didier Trono, via Addgene	RRID: Addgene_12260
pMD2.G	Didier Trono, via Addgene	RRID: Addgene_12259
pRRL.PPT-SFFV-TF plasmids (REPROcode library)	Kurochkin *et a*l. 2026^1^	N/A
pRRL.PPT-SFFV-dTomato	Ascic *et al.* 2024^14^	N/A

**Software and algorithms**		

FlowJo v10	Becton, Dickinson	https://flowjo.com
ImageJ	Schneider *et al.* 2012^15^	https://imagej.net
TF combination identification	Kurochkin *et al.* 2026^1^	Zenodo: https://doi.org/10.5281/zenodo.17348409
TF barcode demultiplexing	Kurochkin *et al.* 2026^1^	Zenodo: https://doi.org/10.5281/zenodo.17348409
STAR v2.7.5	Dobin *et al.* 2013^16^	https://github.com/alexdobin/STAR
BD Rhapsody Sequence Analysis Pipeline v2.2.1	BD Biosciences	https://www.bdbiosciences.com/en-sg/products/software/rhapsody-sequence-analysis-pipeline
Seurat v4.3.0	Hao *et al.* 2021^17^	https://satijalab.org/seurat/
R v4.2.3	R Core Team	https://www.r-project.org

**Deposited data**		

HEFs single-cell RNA-seq barcoded dataset	Kurochkin *et al.* 2026^1^	GEO: GSE298471
YUMM1.7 single-cell RNA-seq barcoded dataset	This paper	GEO: GSE321717
Human blood myeloid cell dataset	Villani *et al.* 2017^18^	GEO: GSE94820

**Other**		

Hemocytometer (Bürker chamber)	Thermo Fisher Scientific	Cat #15501011
MicroAmp™ 8-Tube Strip, 0.2 mL	Thermo Fisher Scientific	Cat #N8010580
BD Rhapsody™ Rhapsody HT Xpress	BD Biosciences	Cat #666625
BD Scanner	BD Biosciences	Cat # 633701
Agilent High Sensitivity DNA Kit	Agilent	Cat #5067-4626
Agilent 2100 Bioanalyzer system	Agilent	Cat # G2939BA
Qubit 4	Invitrogen	Cat #Q33240
Qubit 1X dsDNA High Sensitivity (HS) Assay Kits	Thermo Fisher Scientific	Cat #Q33230

## Materials and equipment

This section provides detailed protocols for buffer and solution preparation.

**Table:** DMEM complete

Reagent	Stock concentration	Amount
DMEM	N/A	500 mL
FBS (heat-inactivated and sterile-filtered)	N/A	50 mL
GlutaMAX	100X	5 mL
Pen-Strep	100X	5 mL

Store DMEM complete at 4°C, up to 1 month.

**Table:** Sorting buffer

Reagent	Stock concentration	Amount
PBS	N/A	500 mL
BSA	10%	2 mL
EDTA	0.5 M	5 mL
Pen-Strep	100X	5 mL

Store sorting buffer at 4°C, up to 1 week.

## Step-by-step method details

### Lentivirus production and titration

**Timing: 7 days**

This section details lentiviral production for the barcoded TF lentiviral library.

**Figure 1:**
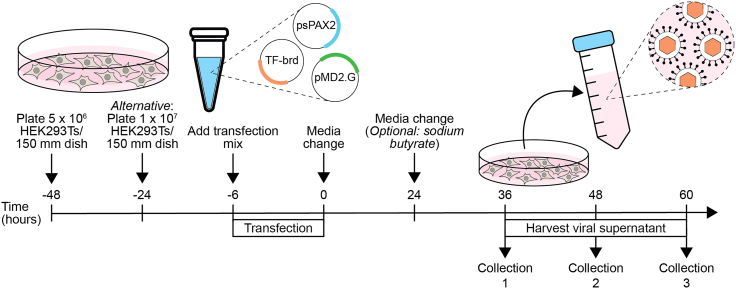
Schematic overview of arrayed lentiviral production Human embryonic kidney 293 T (HEK293T) cells are seeded and transfected with second-generation lentiviral packaging (psPAX2) and envelope (pMD2.G) plasmids along with a barcoded transcription factor (TF-brd) in a lentiviral backbone. The supernatant containing viral particles is collected at three timepoints.

1. Expand the HEK293T cell cultures.
  a. Culture HEK293T cells in 150 mm cell culture treated dishes in DMEM complete and incubate at 37°C with 5% CO_2_.
  b. Change media every 2 days.
  c. Passage cells when they reach ∼80% confluence.
  d. To passage cells, aspirate media and gently rinse with 5 mL of PBS.
  e. Remove PBS and add 5 mL of TrypLE.
  f. Incubate at 37°C with 5% CO_2_ until cells detach with agitation.
  g. Neutralize TrypLE by adding an equal volume of DMEM complete.
  h. Resuspend cells by pipetting and transfer to a conical tube.
  i. Centrifuge at 350 × *g* for 5 min.
  j. Remove supernatant and resuspend the cell pellet in DMEM complete.
  k. Prior to seeding cells for transfection, mix 10 μL of cell suspension with Trypan Blue, load it onto a hemocytometer, and count viable HEK293T cells.
  l. Seed 5 x 10^6^ HEK293T cells per 150 mm cell culture-treated dish in 20 mL of DMEM complete, 48 h before transfection (Figure 1).

**Figure 2:**
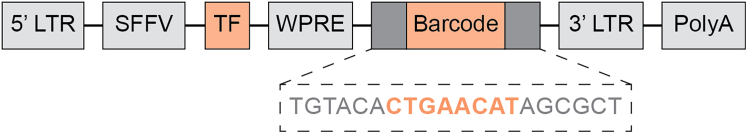
Design of individual barcoded lentiviral vectors Schematic of a lentiviral vector encoding a transcription factor (TF) and corresponding 20-bp barcode sequence. An example of unique 8-bp barcode (orange) flanked by 6-bp constant regions (gray) is shown. Viral elements are indicated in gray boxes, including 5′ and 3′ long terminal repeats (LTR), the constitutive splenic focus-forming virus (SFFV) promoter, the woodchuck hepatitis virus post-transcriptional regulatory element (WPRE), and the poly(A) sequence. The barcode is optimally positioned approximately 300 bp upstream of the poly(A) tail to enable detection by 3′ single-cell mRNA sequencing.

***Note:*** For plating 24 h prior to transfection, seed 1 x 10^7^ HEK293T cells per 150 mm dish.

***Note:*** To prepare arrayed libraries for screening experiments, prepare 1-2 x 150 mm dishes per lentiviral vector carrying an individual barcoded TF (Figure 2).

2. Transfect the HEK293Ts and collect the lentiviral supernatants.
  a. Check HEK293T cells are ≥70% confluent under a light microscope (Figure 3A).
    ***Note:*** Cells should grow as a monolayer and not in clusters, which indicates stress.

**Figure 3:**
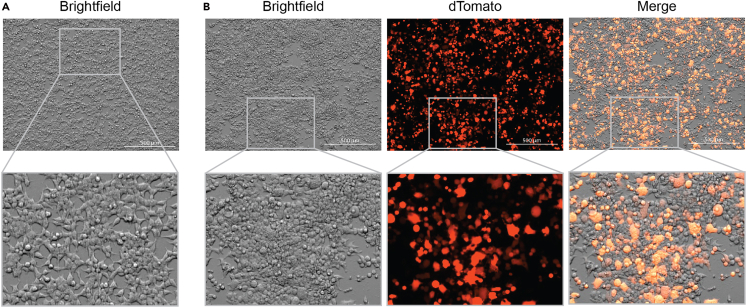
Representative morphology of HEK293T cells during lentivirus production (A) HEK293T cells are 70% confluent at the time of transfection. (B) The majority of transfected cells express dTomato at 60 h post-transfection. Scale bar - 500 μm.
  b. Transfect HEK293T cells.
    i. In a 15 mL conical tube, combine 7.5 μg of supercoiled psPAX2, 2.5 μg of pMD2.G, and 10 μg of the transfer plasmid containing the barcoded TF (e.g., SFFV-PU.1-Brd 1) per 150 mm dish.
    ii. Adjust the volume to 1 mL per dish with Opti-MEM.
    iii. Add 60 μL of the transfection reagent Polyethylenimine (PEI; 1 mg/mL) per dish.
    iv. Mix thoroughly by pulse-vortexing.
    v. Incubate for 15 min at 20°C.
      **CRITICAL:** Add PEI last at a 1:3 ratio to promote PEI-DNA complexes and improve DNA delivery to the nucleus, thereby enhancing transfection efficiency.
    vi. Replace the HEK293T media with 14 mL of DMEM without FBS or antibiotics.
    vii. Add the transfection mix dropwise using a P1000 pipette. The final volume in the dish is 15 mL.
      ***Note:*** When preparing transfection mixes for multiple transfer plasmids in parallel, first prepare a master mix of Opti-MEM, psPAX2, and pMD2.G. Then, aliquot 1 mL of master mix per 150 mm dish in a new 15 mL conical tube and add the transfer plasmid.
    viii. Carefully rotate the dish side-to-side to distribute the mix evenly. Refrain from circular mixing to avoid the transfection mix from congregating in the middle of the dish.
    ix. Incubate cells with the transfection mix at 37°C with 5% CO_2_ for 6 h.
    x. After 6 h, replace the media with 20 mL of DMEM complete.
      ***Note:*** This time point is considered 0 h of the viral collection protocol.
  c. Harvest the lentiviral supernatant.
    i. At 24 h post-transfection, replace the media with 12 mL of fresh DMEM complete.
      ***Optional:*** To enhance lentiviral titers, supplement DMEM complete with sodium butyrate (1 mM), a histone deacetylase inhibitor, which opens chromatin and facilitates transcription of viral components.
    ii. At 36 h, harvest lentiviral supernatants in 50 mL conical tubes (Collection 1) and store at 4°C.
    iii. Replace media with 12 mL of fresh DMEM complete. Repeat this step at 48 h post-transfection (Collection 2).
    iv. Assess transfection efficiency using a control vector with a fluorescent reporter (e.g., dTomato) via fluorescence microscopy (Figure 3B).
      ***Note:*** The lentiviral constructs used to build the REPROcode TF library do not contain dTomato or any other fluorescent reporter.
    v. Collect the final supernatant at 60 h (Collection 3).
    vi. Combine viral supernatants from all collections, approximately 36 mL.
    vii. Discard HEK293T cells according to institutional BSL-2 cell culture laboratory guidelines.
      ***Note:*** This step describes the production of lentivirus encoding individual barcoded TFs. Due to its location in the lentiviral plasmid, the barcode can be read directly using any sequencing technology that relies on 3′ polyA amplification, such as 10X Genomics and BD Rhapsody. To produce lentiviral particles carrying individual barcoded TFs, the transfection uses second-generation lentiviral packaging (psPAX2) and envelope (pMD2.G) plasmids, in addition to the transfer plasmid. Individually produced lentiviruses are pooled and co-transduced to generate an arrayed TF library and used for reprogramming screens.
3. Concentrate the lentivirus.
  a. Filter pooled lentiviral supernatants through a low protein binding polyethersulfone (PES) 0.45 μm filter into a new 50 mL tube.
  b. Add 1 volume of Lenti-X Concentrator per 3 volumes of supernatant (e.g., 12 mL of Lenti-X Concentrator to 36 mL of supernatant).
    ***Note:*** Takara Bio’s Lenti-X Concentrator provides a rapid and scalable method of concentration for processing variable volumes of supernatant and several individual viral preparations at the same time. Alternatively, lentivirus can be concentrated by ultracentrifugation.
  c. Mix by inversion and incubate at 4°C for 30 min to 24 h.
  d. Centrifuge at 1,500 × *g* for 45 min at 4°C.
  e. Aspirate the supernatant without disturbing the pellet, which should be white and visible.
  f. Resuspend the pellet in ice-cold PBS at 1/100 of the original supernatant volume. Work quickly and keep the tubes on ice to maintain viral titers.
  g. Prepare aliquots of 10–20 μL of the resuspended lentivirus into sterile 0.2 mL 8-Tube Strips.
  h. Store aliquots long-term at −80°C.
    **CRITICAL:** Lentivirus aliquots stored at −80°C should not undergo freeze/thaw cycles. Repeated freezing reduces viral titers, affecting TF distribution in arrayed library sequencing data.
4. Titer the lentivirus.
  This step describes the extraction and quantification of viral RNA genome content.
  a. Thaw one aliquot of each lentivirus on ice.
  b. Extract viral RNA using the NucleoSpin RNA Virus Kit, according to the manufacturer’s instructions. Scale kit volumes according to viral aliquot size.
    ***Note:*** The NucleoSpin RNA Virus Kit is used to extract RNA and remove contaminants; alternatively, lentiviral RNA can be extracted using TRIzol Reagent.
  c. To remove any residual plasmid DNA, treat RNA with DNase I, following the Lenti-X qRT-PCR Titration Kit User Manual (Cat. No. 631235 (050819), pages 5–8).
    ***Note:*** Takara Bio’s Lenti-X qRT-PCR Titration Kit is used to perform DNaseI treatment and RT-PCR to quantify viral RNA genome copy number. Because the REPROcode TF library does not contain fluorescence or antibiotic resistance markers, functional tittering is not feasible, therefore, physical tittering with the Lenti-X qRT-PCR Titration Kit is recommended.
    ***Optional:*** Instead of serially diluting viral RNA (see Lenti-X qRT-PCR Titration Kit User Manual, page 7, step 5), use 3–4 technical replicates of undiluted RNA.
  d. Perform qRT-PCR for each lentivirus, according to the Lenti-X qRT-PCR kit manual.
  e. Generate a standard curve by plotting the Cycle Threshold (Ct) values from the Lenti-X RNA control template versus the copy number in log scale.
  f. Calculate average Ct values from the technical replicates of each lentivirus sample.
  g. Using the equation from the standard curve, calculate total RNA copy numbers.
    ***Note:*** Expected titers for Lenti-X concentrated barcoded TF lentiviruses are 10^6^–10^8^ copies/μL.
    **Pause point:** Concentrated lentivirus can be stored at −80°C until the time of use.

### Expansion of human embryonic fibroblasts

**Timing: 2 days**

This section describes the maintenance of human fibroblasts in culture for downstream reprogramming experiments.

5. Expand HEFs.
  a. Pre-coat 100 mm cell culture treated dishes with 5 mL of 0.1% porcine gelatin.
  b. Incubate at 37°C with 5% CO_2_ for 15 min up to 24 h.
  c. Prior to plating, aspirate the gelatin from the pre-coated dishes.
  d. When thawing cryopreserved HEFs, thaw quickly in a 37°C water bath.
  e. When a small frozen pellet is still visible, transfer cells to a 15 mL conical tube containing 10 mL of pre-warmed DMEM complete.
  f. Centrifuge cells at 350 × *g* for 5 min.
  g. Aspirate the supernatant and resuspend the cell pellet in fresh DMEM complete.
  h. Culture HEFs in DMEM complete and incubate at 37°C with 5% CO_2_.
  i. Change media every 2 days.
  j. Passage cells when they reach ∼80–90% confluence.
  k. To passage cells, aspirate culture media and gently rinse cells with 5 mL of PBS.
  l. Remove PBS and add 3–5 mL of TrypLE.
  m. Incubate at 37°C with 5% CO_2_ for 10 min or until cells begin to detach with agitation.
  n. Neutralize TrypLE with an equal volume of DMEM complete and resuspend cells by pipetting.
  o. Transfer to a conical tube and centrifuge at 350 × *g* for 5 min.
  p. Remove the supernatant and resuspend the cell pellet in fresh DMEM complete.
    ***Note:*** HEFs can be cultured for up to 16 passages. For sequencing experiments, use HEFs with fewer than 10 passages.
  q. Plate HEFs in 10 mL DMEM complete and gently rotate the dish to evenly distribute the HEFs.
  r. Incubate at 37°C with 5% CO_2_ until further use.
    ***Note:*** Depending on cell confluence and passage number, a 100% confluent 100 mm dish generally yields 1–3 x 10^6^ HEFs. Splitting ratios can range from 1:4 to 1:6 when plating 2.5–5 x 10^5^ cells per 100 mm dish.
    ***Note:*** HEFs grow as a homogenous, adherent cell population, and are widely used in reprogramming practices due to relatively high plasticity and easy maintenance.

### Determining the viral copy number per cell

This section describes how to optimize the viral copy number for reprogramming experiments.

6. Determine the cp/cell.
  a. Using the RNA copy number per μL (see Step 4g), calculate the required volume of each lentivirus.

**Figure 4:**
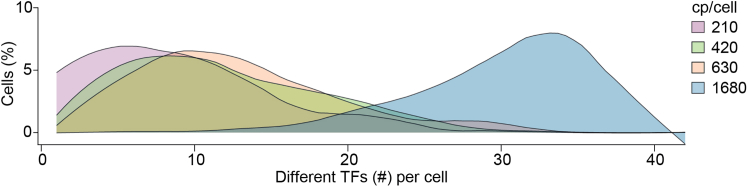
Transcription factor distribution and mode at reprogramming day 9 Distribution of the number of different TFs per single reprogrammed (CD45^+^) cell using a 42-TF pool at a cp/cell of 210 (purple), 420 (green), 630 (orange), or 1680 (blue).

***Note:*** To calculate the volume of virus required for transduction, three values are needed: the number of cells seeded, the desired cp/cell, and the physical titer of each lentivirus. For example, if 4 x 10^5^ HEFs are seeded on a 100 mm dish and the target cp/cell is 400 (e.g., a pool of 40 TFs at 10 cp/TF), the total RNA copies required per TF is 4 x 10^6^ copies = 4 x 10^5^ cells x 10 cp/TF. If the titer of the barcoded TF PU.1 lentivirus is 4 x 10^6^ copies/μL, the required volume of that virus is 1.0 μL = 4 x 10^6^ total copies ÷ 4 x 10^6^ copies/μL. Repeat this calculation for each individual lentivirus in the pool. For calculated volumes less than 1.0 μL, prepare a 1:10 dilution of the stock lentivirus to minimize pipetting error.

**CRITICAL:** Viral copy number is defined as the total viral RNA copies per cell (cp/cell). In combinatorial TF screens that employ arrayed pools of TFs, the optimal cp/cell depends on the number of TFs in the pool.^1^ Although the relationship between the number of TFs included in a pool and the optimal cp/cell is nonlinear, pools with more TFs require lower cp/cell, whereas pools with fewer TFs require a higher cp/cell. Too high cp/cell in large pools (>40 TFs) leads to oversaturation of TFs, which reduces the ability to identify meaningful combinations. For the cDC1 reprogramming system, we showed that a pool of 9 TFs requires 360 cp/cell (40 cp/TF), whereas a pool of 42 TFs requires 420 cp/cell (10 cp/TF).^1^ While the total RNA copies delivered per cell remains similar across these conditions, the cp/TF is reduced as pool size increases.

**CRITICAL:** We have observed that successful reprogramming requires combinations of 3–5 different TFs.^13^ Adjust the cp/cell as needed to reach an optimal modal integrated TF range in successfully reprogrammed cells by day 9 (Figure 4).

### Fibroblast transduction and reprogramming

**Timing: 10 days**

This section outlines the transduction of HEFs with an arrayed, barcoded TF lentiviral library for reprogramming.

7. Seed HEFs for transduction.
  a. Pre-coat 100 mm cell culture treated dishes or 6-well plates with gelatin and incubate at 37°C with 5% CO_2_ for at least 15 min.
  b. Remove DMEM complete from HEF cultures.
  c. Wash cells with PBS.
  d. Incubate with TrypLE for 10 min.
  e. Once cells detach, neutralize TrypLE with DMEM complete.
  f. Transfer the suspension to a conical tube.
  g. Centrifuge at 350 × *g* for 5 min.
  h. Aspirate the supernatant and resuspend the cell pellet in DMEM complete.
  i. Prior to seeding cells for transduction, mix 10 μL of cell suspension with Trypan Blue, load it onto a hemocytometer, and count viable HEFs.
  j. Seed 4 x 10^5^ HEFs per 100 mm (or equivalent density for 6-well plates).
    ***Note:*** Seeding density may vary between 2.5–5 x 10^5^ HEFs per plate, depending on the total cp/cell. For high cp/cell (>300), seed higher cell numbers (≥4 x 10^5^ HEFs per plate). Optimize the seeding density when applying this protocol to other cell types.
  k. Incubate at 37°C with 5% CO_2_ for 2–4 h until cells attach.
8. Transduce HEFs with arrayed lentiviral library.
  a. Thaw aliquots of concentrated lentivirus on ice.
  b. Prepare a master mix of DMEM complete supplemented with polybrene (8 μg/mL).
  c. Add each individual lentivirus according to its corresponding TF. The final volume of the master mix should be 6 mL per 100 mm plate or 1 mL per well in a 6-well plate.
  d. Aspirate the media from HEF-seeded plates.
  e. Add the transduction master mix dropwise.
  f. Carefully rotate the plates side-to-side to distribute the mix evenly.
  g. Incubate at 37°C with 5% CO_2_ for 8–12 h.
  h. Replace media with fresh DMEM complete.

**Figure 5:**
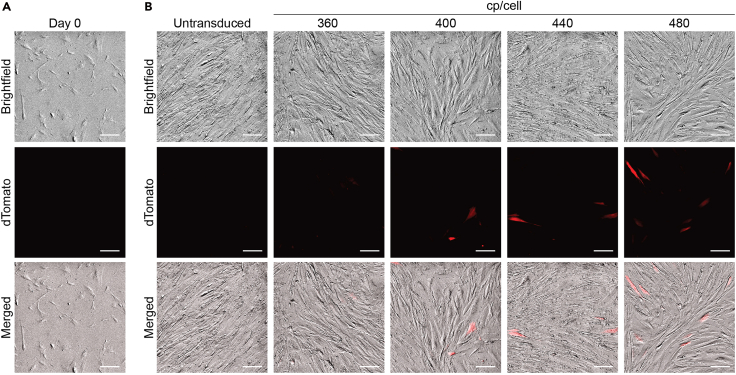
Representative morphology of human embryonic fibroblasts transduced with lentivirus (A) Density of human embryonic fibroblasts at reprogramming day 0 after seeding 4 x 10^5^ cells per 100 mm dish. (B) Human embryonic fibroblasts transduced with lentiviruses expressing dTomato at the indicated total viral RNA copies per cell (cp/cell), 9 days post-transduction. Scale bar - 200 μm.

***Note:*** This time point is considered reprogramming day 0 (Figure 5A).

***Note:*** Because HEFs do not express the hematopoietic marker CD45 or the antigen-presenting marker HLA-DR, they are a valuable starting population for reprogramming towards immune cell fates.

9. Reprogram HEFs.
  a. Replace media every 2 days throughout the reprogramming time course.
  b. Observe cells under an inverted microscope every few days for reprogramming-related death. See troubleshooting problem 1.

**Figure 6:**
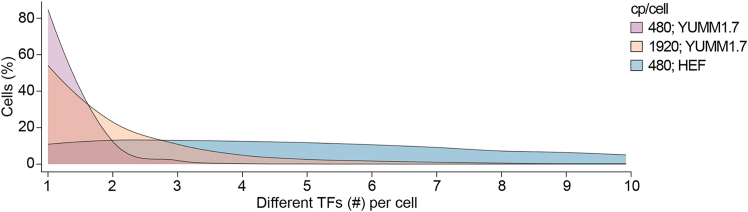
Transcription factor distribution in YUMM1.7 melanoma cells Distribution of the number of distinct TFs per cell measured 48 h after transduction with a 48-TF pool. Cells were transduced at a cp/cell of 480 in YUMM1.7 (purple) and HEFs (blue), and at a cp/cell of 1920 in YUMM1.7 (orange). The range of distribution is shown between 1–10 TFs per cell.

***Note:*** Transduction levels can be assessed using control lentivirus expressing a fluorescent reporter (e.g., dTomato) across different cp/cell (Figure 5B).

**CRITICAL:** Because transduction efficiency varies across cell types, perform a pilot experiment when applying this protocol to a new cell type to determine the appropriate cp/cell. Both different cell types and different passages of the same cell line can exhibit variable permissiveness to lentiviral transduction. Therefore, test a range of cp/cell values to identify the lowest cp/cell that yields sufficient reprogramming efficiency to purify reprogrammed populations. For example, in a pool with 30 immune TFs, if 2% CD45^+^ cells are obtained with 300 cp/cell and 8% with 750 cp/cell, it is recommended to proceed with 300 cp/cell. Sequence live cells at single-cell resolution 48 h post-transduction to assess TF representation. Cell types that are more difficult to transduce, like primary cells, or that exhibit higher proliferation rates, such as YUMM1.7 melanoma cells, may show a reduced distribution of 1–3 TFs per cell at the same cp/cell compared with HEFs (Figure 6).

### Purification of reprogrammed cells

**Timing: 6 h**

This section details the purification of reprogrammed cells by flow activated cell sorting (FACS) prior to library preparation and single-cell RNA sequencing.

**Figure 7:**
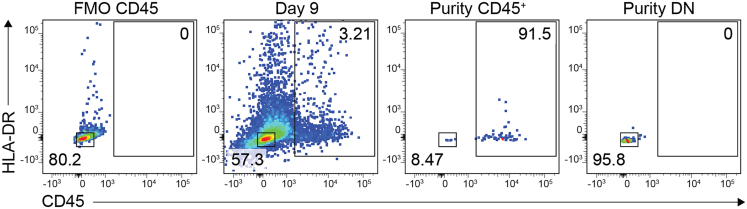
Representative flow cytometry plots for gating strategy Flow cytometry quantification of cell populations FACS-sorted for single-cell RNA sequencing at reprogramming day 9. The CD45 gate was defined using a fluorescence minus one (FMO) control. Purity of the reprogrammed CD45^+^ and non-reprogrammed, double-negative (DN; CD45^-^HLA-DR^-^) populations was assessed. The percentages of cells in each gate are indicated.
  d. Sort a minimum of 1 x 10^4^ reprogrammed cells (CD45^+^) into chilled 1.5 mL Eppendorf tubes pre-coated with 250 μL of sorting buffer.

10. Stain reprogrammed cells with surface markers.
  a. At the reprogramming day endpoint (e.g., day 9 for induced immune cells), remove DMEM complete from plates containing reprogrammed cells.
  b. Wash with PBS.
  c. Incubate with TrypLE for 10 min.
    ***Note:*** While cells reprogrammed from HEFs acquire immune-like morphologies, they remain adherent during the reprogramming process.
  d. Once cells are detached, neutralize with ice-cold sorting buffer.
  e. Transfer to a conical tube.
  f. Centrifuge at 350 × *g* for 5 min.
  g. Aspirate the supernatant and resuspend the cell pellet in sorting buffer at a density of 1 x 10^6^ cells/100 μL.
  h. Transfer to a FACS-compatible 5 mL polystyrene round-bottom tube.
  i. Incubate the cell suspension with mouse serum (1:100) and the appropriate antibody (e.g., CD45) for 30 min at 4°C in the dark.
  j. Wash cells with sorting buffer.
  k. Store cells at 4°C in the dark until sorting.
    ***Optional:*** Reserve an aliquot of the cell suspension to stain for lineage-specific markers (in addition to CD45) to assess whether the lineage of interest is present in the population. Even if present at low frequencies, this indicates that the TF pool contains the reprogramming factors that specify the lineage of interest.
11. Purify reprogrammed cells by FACS.
  a. Use an appropriate cell sorter (e.g., BD FACS AriaIII) with a 100 μm nozzle.
  b. Set gates to exclude doublets and dead cells.
  c. Use a fluorescence minus one (FMO) control to define the CD45^+^ population (Figure 7). See troubleshooting problem 2.

**CRITICAL:** Sort CD45^-^HLA-DR^-^ non-reprogrammed cells in parallel as a control to normalize detection of TF combinations.

12. Count reprogrammed cells after sorting.
  a. Centrifuge the Eppendorf tubes at 350 × *g* for 5 min at 4°C.
  b. Carefully aspirate the supernatant.
  c. Resuspend the cell pellet in BD Sample Buffer.
  d. Count cells using a hemocytometer and a viability stain (e.g., Trypan blue).

***Note:*** Sorting efficiency varies based on cell type and FACS instrument. Do not rely on the sort count reported by the instrument. When sorting HEFs on a BD AriaIII, expect a recovery rate of approximately 60% of the sort count.

### Single-cell capture and cDNA synthesis

**Timing: 4 h**

This section describes single-cell capture and cDNA synthesis using the BD Rhapsody HT-Xpress System.

13. Use quantified cell counts to determine the number of cells to load per lane of the BD Rhapsody cartridge.

**Figure 8:**
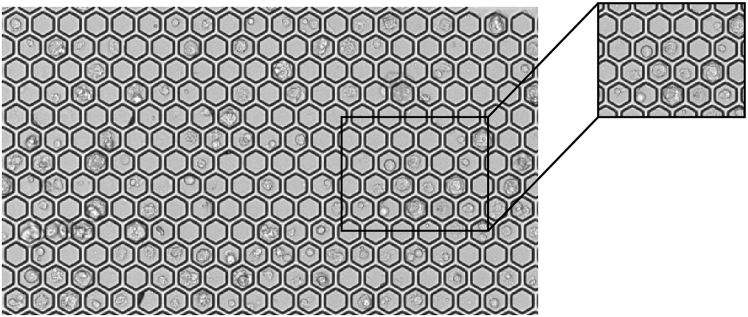
Representative image of BD Rhapsody cartridge microwells acquired using the BD Scanner Cells are seeded in hexagonal microwells after cell loading. A magnified view of human embryonic fibroblasts of varying sizes occupying individual microwells is shown to the right.

***Note:*** The BD Rhapsody platform uses a gravity-based microwell cartridge to capture single-cells, in contrast to microfluidic bead encapsulation methods. Each cartridge processes up to eight samples in parallel. Follow the BD Rhapsody HT Xpress System Protocol (BD Biosciences, Document 23–24253(02), pages 9–19) to prepare the cartridge, load cells with capture beads, perform cell lysis and bead retrieval, perform reverse transcription, and treat beads with Exonuclease I.The Rhapsody 8 Lane Cartridge is designed for up to 1.0 x 10^5^ hematopoietic cells per lane on current models. Larger reprogrammed fibroblasts (>40 μm), may be loaded at approximately 5.5 x 10^4^ cells per lane to account for size-related cell loss. Increasing the number of cells loaded may increase the doublet rate, therefore, loading conditions for each cell type should be optimized. Optimization can be performed using a previously used, washed cartridge by loading a range of cell numbers across lanes and evaluating both the number of cells successfully captured and the doublet rate. This assessment can be conducted using the BD Rhapsody Scanner or standard light microscopy.

***Optional:*** When testing cell capture rate for the first time, it is recommended to visualize the loaded cartridge using the BD Scanner to estimate cell capture efficiency and doublet rate (Figure 8). Expected capture and doublet rates for HEFs range between 50–70% and 15–25%, respectively.

**Pause point:** Exonuclease I-treated beads can be stored at 4°C for up to one year before proceeding with library preparation.

### Library preparation

**Timing: 12 h**

This section describes the preparation of the mRNA gene expression library.

**Figure 9:**
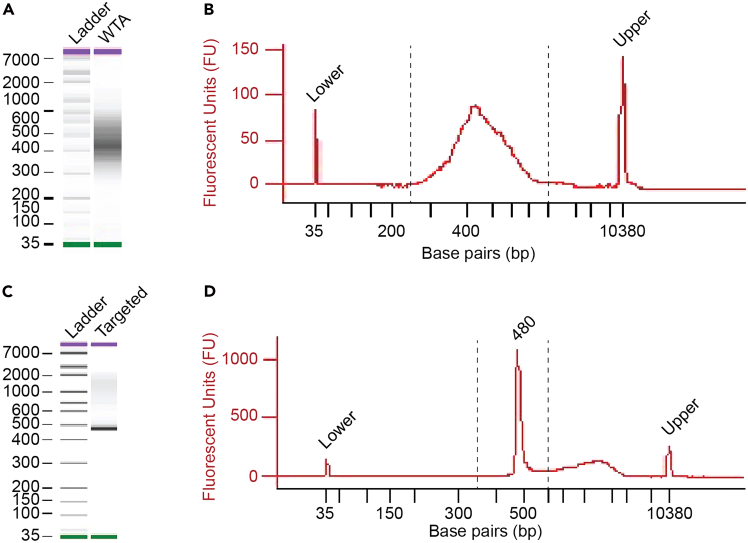
Representative bioanalyzer trace of library Quality control of the final whole transcriptome analysis (WTA) index PCR product (A and B) and mRNA targeted index PCR product of the barcode targeted library (C and D) on gel and electropherogram. Upper (purple) and lower (green) ladder peaks are indicated; dashed lines show ranges for average fragment size measurements: 250-1000 bp for WTA and 350–600 bp for targeted.

14. Prepare mRNA gene expression libraries by following the BD Rhapsody mRNA WTA and Sample Tag Library Preparation Protocol (BD Biosciences, Document 23–24117(03), pages 13–27) with the following modifications:
  a. Repeat the WTA index PCR product purification step (dual-sided cleanup) for a total of two rounds, see section titled, Additional WTA index PCR cleanup (page 25).
  b. Quantify the WTA index PCR products with a Qubit™ Fluorometer using the Qubit™ dsDNA High Sensitivity Assay.
  c. Assess library quality control using an Agilent 2100 Bioanalyzer with the Agilent High Sensitivity DNA Kit.
  d. On the bioanalyzer trace, expect amplified transcript fragments within a size range of 250–100 base pairs (Figures 9A and 9B).

**Table 1:** Oligonucleotide sequences for targeted PCR

Use	Primer name	Sequence (5′ to 3′)
PCR1	N1	CTTTCTGGGACTTTCGCTTTCCCC
PCR2	N2	TTGCAATTCGATATCAAGCTGTACCTTCTAGA

PCR1 and PCR2 primer sequences for targeted mRNA library preparation. N2 primer specifically amplifies the targeted barcoded region on the PCR1 product.

***Note:*** Preparation of whole transcriptome analysis (WTA) libraries allows individual barcoded TFs to be directly linked to individual cells expressing gene signatures of interest. This sequencing strategy maximizes the outcomes of the REPROcode platform, including optimal TF stoichiometry, fidelity-enhancing reprogramming factors, and drivers of cell states. In this step, the BD Rhapsody WTA Amplification Kit is used to generate random-primed extension products from the BD Rhapsody Enhanced Cell Capture Beads used in Step 13, followed by PCR amplification and subsequent addition of sequencing adapters and indexing primers to produce sequencing-compatible mRNA libraries.

***Optional:*** Sequencing targeted libraries only provides information on TF representation and distribution per single-cell but does not capture gene expression data. Therefore, TF combinations cannot be linked to target cell types or specific transcriptional states. This option is most useful for cost-efficient pilot experiments aimed at optimizing cp/cell. WTA and targeted amplification library preparations can also be used in tandem if the WTA sequencing depth does not sufficiently capture the barcode information; in this case, targeted amplification can be performed to recover TF barcode data. For targeted barcode amplification, after the two rounds of RPE on BD Rhapsody Enhanced Cell Capture Beads, use the REPROcode Custom Primer Panel (Table 1) and follow the BD Rhapsody System TCR/BCR Full Length and Targeted mRNA Library Preparation Protocol (BD Biosciences, Document 23–24013(03), pages 12–23). Follow the steps only for the Targeted mRNA panel and use the BD Rhapsody Targeted mRNA and AbSeq Amplification Kit to perform a two-step nested amplification of the barcoded region of the integrated lentiviral plasmid. Apply the following modifications: perform 10 PCR cycles for PCR1 and perform quality control on the final sequencing libraries using an Agilent 2100 Bioanalyzer. The expected peak range for the targeted index PCR product is 350–600 bp, with a strong peak at 480 bp, confirming amplification of the barcoded region (Figures 9C and 9D).

**Pause point:** Indexed libraries can be stored at −20°C for up to six months before proceeding with sequencing.

### Sequencing of libraries

**Timing: 24 h**

This section describes pooling and sequencing settings for the mRNA WTA library.

15. Pool equimolar WTA index PCR libraries from all samples to achieve a final concentration of 2 nM.
16. Sequence the libraries to achieve a sequencing depth of ∼70,000 reads per cell.

***Note:*** Barcode detection is compatible with both the 10X Genomics and BD Rhapsody platforms, and the downstream analysis is platform-agnostic.

***Note:*** Libraries are compatible with modern Illumina instruments (e.g., NovaSeq X System, NovaSeq 6000 System).

***Note:*** The BD Rhapsody library preparation kit includes four unique 3′ indexing primers and one common 5′ primer. To increase the number of libraries sequenced, use BD Rhapsody and Illumina compatable indexes (e.g. TruSeq combinatorial dual index primers; Document ID: 23-22747-00)

**CRITICAL:** Do not overload the cartridge. A minimum of 50,000 reads per cell is required for sufficient capture of barcoded sequences. Oversaturation of the cartridge will lead to insufficient sequencing depth.

***Optional:*** Perform this step to sequence only the targeted amplification of the barcoded region. This approach yields information only on TF representation and distribution per single-cell and not gene expression data. To sequence the targeted mRNA library, load the library with 30% PhiX onto a cartridge of at least 200 cycles. Sequence using paired-end sequencing mode with a read structure of 85-8-8-135.

### Data analysis

**Timing: 3 days**

This section describes the computational workflow for key analytical steps, including data pre-processing, pipeline setup, quality control, normalization, clusturing, and TF combination discovery. Total analysis timing varies based on sequencing depth, analysis complexity, and the number of samples and cells.

17. Pre-process the FASTQ files using SevenBridges.
  a. Create a new account and project on the BD SevenBridges platform (http://www.sevenbridges.com/bdgenomics/).
  b. Upload the FASTQ files to the project.
    ***Note:*** Perform file uploads via the command line (https://docs.sevenbridges.com/docs/upload-via-the-command-line).
  c. Add the BD Rhapsody Sequence Analysis Pipeline App (v3.0) to the project.
  d. Generate a new task by selecting “Run” in the “Apps” tab.
  e. Open the newly created task and add the FASTQ read files (both R1 and R2) to the “Reads” tab.
  f. Add the desired reference genome files to the “Reference Files Archived”.
  g. Open the App Settings and select “Misc_Settings”.
  h. Set “Exclude Intronic Reads” to *False.*
  i. Under “Bam_Settings,” set “Generate Bam Output” to *True.*
  j. In the App Settings, under “Putative_Cell_Calling_Settings”, set “Expected Cell Count” to the expected cell count (e.g., 20,000 cells).
    ***Note:*** The output Bam file contains the aligned reads to the reference genome as well as non-aligned reads that contain the barcoded sequence.
    **CRITICAL:** Review the SevenBridged HTML report after pipeline completion. Assess the quality of the data by examining the putative cell number and the mean reads per cell for each sample. Expect a minimum cell recovery rate of 20%. If skipping the barcode amplification step, ensure a mean sequencing depth greater than 50,000 reads per cell.
18. Detect the TF barcodes using the previously downloaded Bam files from SevenBridges.
  a. Install Conda and Mamba.
  b. Download REPROcode pre-processing tools (DOI: https://doi.org/10.5281/zenodo.17348409).
  c. Install the required dependencies:
    mamba env create -n REPROcode -f environment.yml
  d. Activate the environment:
    conda activate REPROcode
  e. Prepare a FASTA file containing the 8-bp variable barcode regions corresponding to each transcription factor included in the experiment.
    Examples of files:
    >SPI1
    CTGAACAT
    >IRF8
    GTTTTTTA
    >BATF3
    TTTCTGTT
  f. Run the barcode detection tool: See troubleshooting problem 3.
    python bcodes.py --barcodes=tf-barcodes.fasta --technology rhapsody ∗_Bioproduct.bam > table.tsv
    Example of output file:
    cell.ID  is.Cell  UMI.adjusted  UMI.raw  TF.barcode  TF.name
    30761441  T  CTCTACGC  CTCTACGC  TTTCTGTT  BATF3
    35577058  T  ACCATCAT  ACCATCAT  TTTCTGTT  BATF3
    22935007  T  TCTTTGTC  TCTTTGTC  GTTTTTTA  IRF8
    11963992  T  GCAATACT  GCAATACT  CTGAACAT  SPI1
    ***Note:*** The barcode detection tool uses the non-aligned fraction of the reads in the Bam file.
19. Pre-process transcriptomic single-cell data.
  a. Load the Seurat object (.rds) generated from the SevenBridges pipeline (step 17f).
    Library(Seurat)
    Seurat.object <- readRDS(“Seurat.rds”)
    Seurat.object[[“percent.mt”]] <- PercentageFeatureSet(Seurat.object, pattern = “ˆMT-“)
  b. Define and apply a function to filter outlier cells based on median absolute deviation (MAD) thresholds.
    is_outlier <- function(Seurat.object, metric, nmads) {
      M <- seurat.object@meta.data[,metric]
      med <- median(M)
      mad_val <- mad(M, constant = 1)
      outlier <- (M < (med - nmads ∗ mad_val)) | (M > (med + nmads ∗ mad_val))
      return(outlier)
    }
    Seurat.object.flt <- Seurat.object[,(!((is_outlier(Seurat.object,“nCount_RNA”,3)) |
    (is_outlier(Seurat.object, “nFeature_RNA”,3)) |
    (is_outlier(Seurat.object, “percent.mt”,3))))]
    **CRITICAL:** Examine the distribution of UMIs, “nCount_RNA,” and filter out cells with <20,000 UMI counts.
  c. Normalize the dataset and perform dimension reduction and data clustering.
    ArrayGEX <- NormalizeData(Seurat.object.flt) %>%
     FindVariableFeatures(nfeatures = 5000) %>%
     ScaleData(features = rownames(Seurat.object.flt)) %>%
     RunPCA(verbose = FALSE) %>%
     RunUMAP(dims = 1:30) %>%
    FindNeighbors(dims = 1:30, verbose = FALSE) %>%
     FindClusters(resolution = 2, verbose = FALSE)
    ***Note:*** To identify informative number of clusters, testing multiple resolutions, generally between 0.6 and 3.0 with 0.2 increments, is recommended. This can later be adjusted based on target gene signatures.
20. Load TF matrices and calculate UMI counts for exogenous TF expression.

 library(dplyr)

 matrixTF <- read.table(table.tsv,sep=“\t”,header=T)

 matrixTF <- matrixTF[matrixTF$TF.barcode.name!= “”,]

 matrixTF.flt <- matrixTF[matrixTF$Cell.barcode %in% colnames(Seurat.object.flt),]

 tf_barcodes <- unique(matrixTF.flt$TF.barcode.name)

 cell_barcodes <- colnames(Seurat.object.flt)

 matrixTF.filled <- matrix(0, nrow = length(tf_barcodes), ncol = length(cell_barcodes))

 colnames(matrixTF.filled) <- as.character(cell_barcodes)

 rownames(matrixTF.filled) <- as.character(tf_barcodes)

 align_summary <- matrixTF.flt %>%

  group_by(TF.barcode.name, Cell.barcode) %>%

  summarise(UMI_count = n_distinct(UMI), .groups = 'drop')

 matrixTF.filled[cbind(align_summary$TF.barcode.name, align_summary$Cell.barcode)] <- align_summary$UMI_count

**Figure 10:**
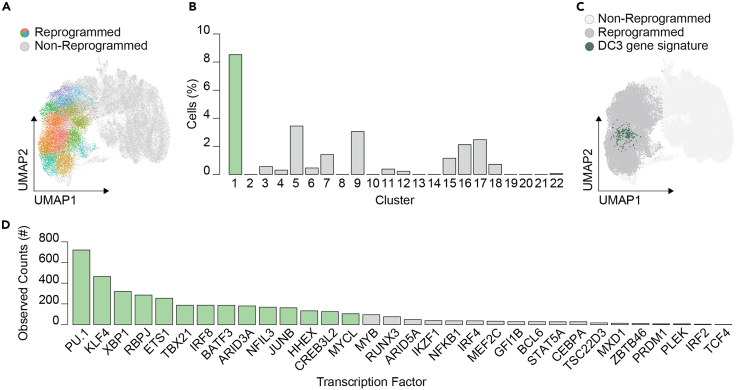
TF frequencies extracted from the DC3 cluster (A) Reprogrammed (CD45^+^) cells visualized on UMAP projection divided into Louvain clusters. Colors indicate distinct clusters, and gray indicates non-reprogrammed cells. Panel reprinted and adapted with permission from Kurochkin *et al*.^1^ (B) Barplot showing the percentage of cells within each cluster expressing a DC3 gene signature.^18^ Green indicates the cluster with the most frequent DC3 signature expression. (C) UMAP visualization of non-reprogrammed (light gray) and reprogrammed (dark gray) cells and cells expressing DC3 gene signature in cluster 1 (dark green). (D) Barplot showing the number of observed counts per TF detected in the top 1000 combinations in cluster 1. Green indicates statistical significance based on permutation test.

21. Examine Louvain clusters for enrichment of pre-defined gene signatures to identify reprogrammed immune cell types (Figure 10A). For example, cluster 1 is enriched in DC3-associated gene signatures,^18^ when compared to 21 other clusters (Figures 10B and 10C).

ArrayGEX <- AddModuleScore(object = ArrayGEX,

         features = list(gene.signature),

         ctrl = 25,

         name = 'DCsign'

)

ArrayGEX$ClassTF = 0

ArrayGEX$ClassTF[ArrayGEX$DCsign > quantile(ArrayGEX$DCsign,0.99)] = 1

ClustEnrich <- table(ArrayGEX$ClassTF,ArrayGEX$RNA_snn_res.2)

ClustEnrich <- ClustEnrich[2,]/table(ArrayGEX$RNA_snn_res.2)∗100

***Note:*** Users must define and apply the relevant gene signature for their target cell type.

**Figure 11:**
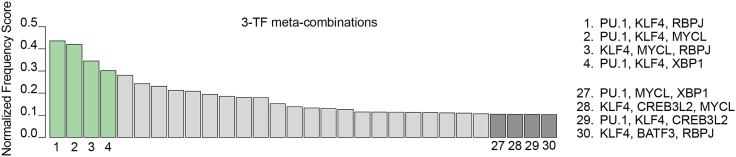
Meta-combinations assembled from DC3 TF frequencies Combinations are ordered by their frequency in reprogrammed (CD45+) cells. The top four most frequent and bottom four least frequent 3-TF meta-combinations in DC3-restricted cells are highlighted.

22. Identify TF enrichment within the specific cluster using custom functions. For example, TFs PU.1, KLF4, XBP1, and RBPJ are among the most frequent TFs detected in the DC3 cluster 1 (Figure 10D).
  a. Load the helper functions from the REPROcode pre-processing tools.
    source(“REPROcode.helper.R”)
  b. Define the parameters for TF identification.
    results.final <- list()
    TFsaved = list()
    Ns   <- c(1000)
    n_perm <- 1000
    alpha  <- 0.05
    cls <- 1
    **CRITICAL:** Define “cls” as the specific cluster corresponding to the target cell type.
    ***Note:*** Users may also adjust: “Ns,” the number of meta-combinations to select from; “n_perm,” the number of permutations used to estimate statistical significance of individual TFs; and “alpha,” the level of significance.
  c. Assemble all meta-combinations and calculate the number of cells containing each combination within the target cluster and within the control population (e.g., CD45^-^HLA-DR^-^ cells).
    res <- build_res(ArrayGEX,matrixTF.filled,cls)
  d. Calculate the frequency of each TF extracted from the Ns meta-combinations (defined in step 22b) and estimate the statistical significance of each individual TF.
    for (N in Ns) {
     message(“ testing top ”, N)
     res_top <- test_topN_parallel(res, N, n_perm = n_perm, alpha = alpha)
     results.final[[paste0(“Clust:”,paste0(cl,collapse = “;”),“;TopCombo:”,N)]] <- res_top
     df <- res_top
     df <- as.data.frame(df[order(df$obs_count,decreasing = T),])
     TFsubs <- as.character(df[df$significant==TRUE,1])
     TFsaved[[“Combo”]] = TFsubs
     }
     plot_tf_counts(res_top, N, cl)
    }
23. Assemble 3 and 4-TF meta-combinations for the desired cell type within the defined cluster using signficantly enriched indivudual TFs and custom REPROcode functions (Figure 11). See troubleshooting problem 4.

rep1 <- process_rep(ArrayGEX,matrixTF.filled,TFsaved$Combo,cls)

print(rep1$res3) # rep1$res3 correspond to 3-TF meta-combinations

print(rep1$res4) # rep1$res4 correspond to 4-TF meta-combinations

**CRITICAL:** Modify “process_rep” function accordingly and specify gene signature of interest. Document all analysis parameters, save intermediate outputs, and validate results across multiple samples.

***Note:*** Complete analysis scripts and parameter files are available in Zenodo archive (https://doi.org/10.5281/zenodo.17348409).

## Expected outcomes

This protocol generates high-quality, single-cell transcriptomic data of diverse induced immune cell populations within a single reprogramming experiment. When performing combinatorial TF screens using REPROcode, several critical and user-defined parameters must be considered during experimental design (Table 2). These include lentiviral library production, transduction and reprogramming, purification of target cell populations, sequencing depth, as well as several user-selected strategies such as target cell type and TF selection.

**Table 2:** Parameters for REPROcode experimental desig**n**

Critical	User-defined
Arrayed lentiviral production and transduction	Whole transcriptome sequencing versus targeted amplification of barcoded sequences
Optimized total viral RNA copies per cell (cp/cell) to achieve appropriate TF distribution per cell	Promoter selection; SFFV is optimal for immune cell reprogramming, but alternative promoters may be used for other cell types
Purification of reprogrammed and non-reprogrammed cell populations	Single-cell capture and library preparation using BD Rhapsody versus 10X Genomics platforms
Sequencing depth of >50,000 reads per cell and >20,000 UMI counts per cell	Selection of target cell type, identification of candidate TFs, and definition of a target cell-specific gene signature

When establishing the REPROcode platform in new cell types, several critical parameters must be optimized, along with additional user-defined experimental choices.

Following transduction with an arrayed, barcoded TF lentiviral library, successfully reprogrammed cells are purified by sorting for CD45 or another lineage-selective marker, while non-reprogrammed or negative control cells are collected to reduce background noise. Whole transcriptome sequencing generates simultaneous gene expression information and detection of exogenous TFs present across cells based on unique barcode detection. Data outputs typically include dimensionality reduction visualization (e.g., UMAPs), cell clustering, module scores, and quantitative summaries of barcoded TF frequencies across cells.

Using these outputs, researchers can identify key TF combinations enriched in cell clusters with distinct transcriptional signatures of specific immune lineages, distinguished from non-reprogrammed cells. Analysis of exogenous TF levels within lineage-affiliated cells informs optimal TF stoichiometry, providing guidance for TF order in polycistronic vector design for subsequent validation experiments. Further interrogation of reprogrammed cells using cell type-specific gene signatures with functional or activated states can inform transcriptional drivers of distinct cell states within a particular lineage. Collectively, REPROcode not only offers a screening platform for combinatorial TF discovery but also identifies stoichiometry requirements, reprogramming fidelity enhancers, and regulators of cell state.

## Limitations

Titration of lentiviruses by qRT-PCR quantifies viral RNA genome copies but does not distinguish the quantity of functional from non-functional viral particles, making it difficult to precisely determine the TF dosage per cell. Although the cp/cell is optimized to achieve a desired range of distinct TFs per cell, this approach does not allow a controlled delivery of an exact number of TFs to each cell. Future technological advances enabling selective profiling of cells that receive 3–5 distinct TFs would simplify screening and reduce sequencing costs. Finally, while meta-combinations provide a practical screening framework, they may not reflect the true combinations present within reprogrammed cells and therefore require subsequent validation.

## Troubleshooting

### Problem 1

High cell death during the reprogramming time course (related to step 9). Excessive cell loss during reprogramming reduces the number of cells available for analysis and may compromise data quality. This can result from unoptimized cell seeding density or too high viral titers.

### Potential solution

Increase the number of seeded cells to ensure sufficient cell numbers at the end of reprogramming while avoiding over-confluence. Alternatively, optimize the arrayed TF pool by either reducing the number of TFs or decreasing the overall cp/cell.

### Problem 2

Surface expression of CD45 (or other marker of interest) cannot be detected by flow cytometry (related to step 11). Enrichment for a reprogrammed population is essential for successfully detecting TF combinations. Low or absent surface marker expression can indicate insufficient cp/cell or suboptimal TF selection.

### Potential solution

Reduce the number of TFs in the pool and increase cp/cell accordingly until sufficient surface marker expression is detected. Alternatively, maintain the overall cp/cell but increase the cp/TF/cell for key TF regulators (e.g., increase cp/TF/cell of the pioneer factor PU.1 to enhance CD45 in hematopoietic reprogramming).

### Problem 3

The barcode sequences cannot be detected (related to step 18). Failure to detect barcode sequences can result from insufficient sequencing depth or high levels of ambient RNA.

### Potential solution

Ensure library is sequenced at an adequate depth (>50,000 reads/cell) and avoid overloading the sequencing cartridge. Retain the WTA library beads to allow targeted barcode amplification at a later stage, if necessary, to recover barcode information.

### Problem 4

Oversaturation of TF combinations (related to step 23). High distribution of the number of TFs per cell can impair the resolution and accuracy of detected TF combinations.

### Potential solution

Reduce the cp/cell to achieve a modal range of 3–5 TFs per cell.

## Resource availability

### Lead contact

Further information and requests for resources and reagents should be directed to and will be fulfilled by the lead contact, Ilia Kurochkin (ilia.kurochkin@med.lu.se).

### Technical contact

Further information and requests for technical questions on executing this protocol should be directed to and will be answered by the technical contact, Abigail R. Altman (abigail.altman@med.lu.se).

### Materials availability

This study did not generate new unique reagents.

### Data and code availability

• scRNA-seq data have been deposited at GEO and are publicly available from the date of publication. Accession numbers are listed in the key resources table.
• This paper analyzes existing, publicly available data. The accession numbers for these datasets are listed in the key resources table.
• This paper does not report original code.
• Additional information required to reanalyze the data reported here is available from the lead contact upon request.

## Acknowledgments

We thank the Center for Translational Genomics and Clinical Genomics Lund, SciLifeLab, for providing sequencing services and the Lund Stem Cell Center FACS facility for cell sorting assistance. This project received funding from the 10.13039/501100000781European Research Council (10.13039/501100000781ERC) under the European Union’s Horizon 2020 research and innovation program (866448-TrojanDC and 101189370-DART). This project was also funded by the 10.13039/100018703European Innovation Council (101130218-RESYNC), 10.13039/501100002794Cancerfonden (23 2932 Pj), the 10.13039/501100004359Swedish Research Council (2020-00615), NovoNordisk Fonden (NNF22C0079466), 10.13039/501100001871FCT (2022.02338.PTDC), and Plano de Recuperação e Resiliência de Portugal pelo fundo NextGenerationEU (grant C644865576-00000005). The 10.13039/501100004063Knut and Alice Wallenberg Foundation, the Medical Faculty at Lund University, and Region Skåne are acknowledged for financial support. I.K. is supported by a Cancer Research Institute Immuno-Informatics Postdoctoral Fellowship (CRI5008). D.P.-C. is supported by an FCT doctoral scholarship (10.54499/2022.15306.BD). The computational resources were provided by the National Academic Infrastructure for Supercomputing in Sweden (NAISS), partially funded by the 10.13039/501100004359Swedish Research Council through grant agreement no. 2022-06725. We would also like to thank Hsiu-Chuan Lin (Centre for Genomic Regulation, Spain) and Lay Teng Ang (Stanford University, USA) for their thoughtful feedback on this protocol, and Mariana Lopes (Cell Reprogramming in Hematopoiesis and Immunity Laboratory) for editing and proofreading.

## Author contributions

A.R.A., C.-F.P., and I.K. were responsible for conceptualization and experimental design. A.R.A. and D.P.-C. performed experiments. I.K. analyzed and interpreted scRNA-seq data and developed barcode detection software. C.-F.P. and I.K. supervised the study. A.R.A., C.-F.P., and I.K. wrote the manuscript. All authors contributed to data interpretation.

## Declaration of interests

C.-F.P. has equity interests and serves in a management position at Asgard Therapeutics AB, which develops cancer immunotherapies based on DC reprogramming technologies. C.-F.P. is an inventor on granted patents U.S. 11,345,891, JP 7303743, and CN ZL201880005047.3 and on patent application WO2018/185709 held by Asgard Therapeutics that cover the cell reprogramming approach described here.
